# Supplementary material for: A Global Screen for Assembly State Changes of the Mitotic Proteome by SEC-SWATH-MS
Source: Cell Syst. 2020 Feb 26;10(2):133–155.e6. doi: 10.1016/j.cels.2020.01.001 (PMC7042714; doi:10.1016/j.cels.2020.01.001)

Q9UHG3 | PCYOX_HUMAN | PCYOX1 KIAA0908 PCL1 UNQ597/PRO1183
Monomer MW [kDa]: 56.64 Monomer expected elution fraction: 45

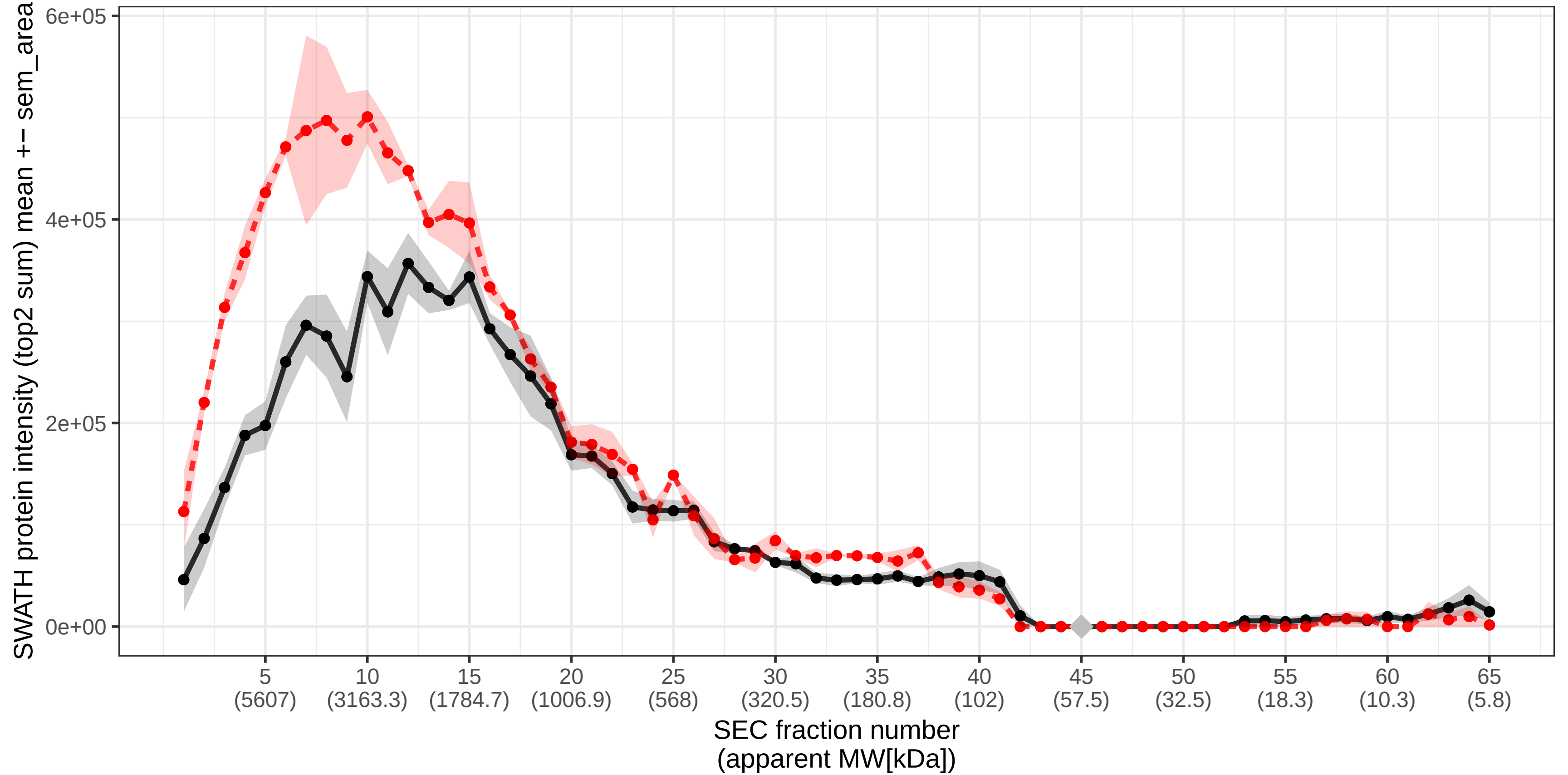

Supplement: Data S1. SEC-SWATH-MS Protein Chromatograms, Related to Figure 1 [file mmc6.zip › SECchrom_Q9UHG3_PCYOX_HUMAN_PCYOX1_KIAA0908_PCL1_UNQ597_PRO.pdf]
